# Supplementary material for: Neural control of growth and size in the axolotl limb regenerate
Source: eLife. 2021 Nov 15;10:e68584. doi: 10.7554/eLife.68584 (PMC8716110; doi:10.7554/eLife.68584)
Supplement: Transparent reporting form [file elife-68584-transrepform1.docx]

***eLife’s* transparent reporting form**

We encourage authors to provide detailed information *within their submission* to facilitate the interpretation and replication of experiments. Authors can upload supporting documentation to indicate the use of appropriate reporting guidelines for health-related research (see [EQUATOR Network](http://www.equator-network.org/%20)), life science research (see the [BioSharing Information Resource](https://biosharing.org/)), or the [ARRIVE guidelines](http://www.plosbiology.org/article/info:doi/10.1371/journal.pbio.1000412) for reporting work involving animal research. Where applicable, authors should refer to any relevant reporting standards documents in this form.

If you have any questions, please consult our Journal Policies and/or contact us: [editorial@elifesciences.org](mailto:editorial@elifesciences.org).

**Sample-size estimation**

- You should state whether an appropriate sample size was computed when the study was being designed
- You should state the statistical method of sample size computation and any required assumptions
- If no explicit power analysis was used, you should describe how you decided what sample (replicate) size (number) to use

Please outline where this information can be found within the submission (e.g., sections or figure legends), or explain why this information doesn’t apply to your submission:

Sample size for in vivo studies were based on sample sizes used in similar studies performed in the Axolotl. For quantitative microscopy-based studies, a minimum of 3 technical replicates were performed for each biological replicate, and a minimum of 3 biological replicates for each sample type were obtained. Details on technical and biological replicates for individual experiments are provided in figure legends and the materials and methods section where appropriate.

**Replicates**

- You should report how often each experiment was performed
- You should include a definition of biological versus technical replication
- The data obtained should be provided and sufficient information should be provided to indicate the number of independent biological and/or technical replicates
- If you encountered any outliers, you should describe how these were handled
- Criteria for exclusion/inclusion of data should be clearly stated
- High-throughput sequence data should be uploaded before submission, with a private link for reviewers provided (these are available from both GEO and ArrayExpress)

Please outline where this information can be found within the submission (e.g., sections or figure legends), or explain why this information doesn’t apply to your submission:

Each biological replicate refers to data that was obtained from a single animal. For example if there are 5 biological replicates, then this data was collected from 5 individual animals. A minimum of 3 technical replicates were performed for each biological replicate. For example, in limb and body length measurements, 3 measurements were obtained for each animal and averaged. For microscopy studies, at least 3 different tissue sections were quantified and averages for each biological replicate. We did not remove any “outliers” from our data. All the data that we obtained was included in our analysis and is provided in the source data files.

Details on technical and biological replicates for individual experiments are provided in figure legends and the materials and methods section where appropriate.

**Statistical reporting**

- Statistical analysis methods should be described and justified
- Raw data should be presented in figures whenever informative to do so (typically when N per group is less than 10)
- For each experiment, you should identify the statistical tests used, exact values of N, definitions of center, methods of multiple test correction, and dispersion and precision measures (e.g., mean, median, SD, SEM, confidence intervals; and, for the major substantive results, a measure of effect size (e.g., Pearson's r, Cohen's d)
- Report exact p-values wherever possible alongside the summary statistics and 95% confidence intervals. These should be reported for all key questions and not only when the p-value is less than 0.05.

Please outline where this information can be found within the submission (e.g., sections or figure legends), or explain why this information doesn’t apply to your submission:

For each quantification when statistical tests were performed, the N, type of test, and p-value are stated in the figure legend. Our methods for statistical analyses were based on previously published analyses on similar experiments in axolotl. In all experiments, the “N” refers to the number of biological replicates. When error bars are present, they represent either the standard deviation or the SEM which is indicated in the figure legend. Exact p values for all data can be found in the source data file for each figure.

(For large datasets, or papers with a very large number of statistical tests, you may upload a single table file with tests, Ns, etc., with reference to sections in the manuscript.)

**Group allocation**

- Indicate how samples were allocated into experimental groups (in the case of clinical studies, please specify allocation to treatment method); if randomization was used, please also state if restricted randomization was applied
- Indicate if masking was used during group allocation, data collection and/or data analysis

Please outline where this information can be found within the submission (e.g., sections or figure legends), or explain why this information doesn’t apply to your submission:

Samples and animals were randomized into groups for each experiment.

**Additional data files (“source data”)**

- We encourage you to upload relevant additional data files, such as numerical data that are represented as a graph in a figure, or as a summary table
- Where provided, these should be in the most useful format, and they can be uploaded as “Source data” files linked to a main figure or table
- Include model definition files including the full list of parameters used
- Include code used for data analysis (e.g., R, MatLab)
- Avoid stating that data files are “available upon request”

Please indicate the figures or tables for which source data files have been provided:

There are source data files containing the information and more specific P values for Figures 1, F1-S2, 2, 3, F3-S1, 4, F4-S1, 5, F5-S1, F5-S2, 7, and 8. Microsoft EXCEl was used to process all data.
